# Supplementary material for: miR526b and miR655 Induce Oxidative Stress in Breast Cancer
Source: Int J Mol Sci. 2019 Aug 19;20(16):4039. doi: 10.3390/ijms20164039 (PMC6720387; doi:10.3390/ijms20164039)
Supplement: Supplementary file 1 [file ijms-20-04039-s001.pdf]

International Journal of
Molecular Sciences

MDPI

Supplementary Materials

A

			MCF7		MCF7-miR526b		MCF7-miR655	
			1	2	5	6	9	10
Key:	**Negative Control**	A	Result 1	Result 2	Result 1	Result 2	Result 1	Result 2
	**Test Group**	C	Result 1	Result 2	Result 1	Result 2	Result 1	Result 2
		E	Result 3	Result 4	Result 3	Result 4	Result 3	Result 4

Calculation: **Total Emission = (Test - Negative)**

B

			Basal		miR526b cond.		miR655 cond.	
			1	2	4	5	7	8
Key:	**Negative Control**	A	Result 1	Result 2	Result 1	Result 2	Result 1	Result 2
	**Test Group**	B	Result 1	Result 2	Result 1	Result 2	Result 1	Result 2
		D			Result 3	Result 4	Result 3	Result 4
		E				Result 3	Result 3	Result 4
		G				Result 4		

Calculation: **Total Emission = (Test - Negative)**

C

			Basal	miR526b cond.	miR655 cond.
			2	6	8
Key:	**Test Group**	A	Result 1	Result 1	Result 1
		B	Result 2	Result 2	Result 2
		C	Result 3	Result 3	Result 3

Figure S1. ROS and SO measurement plan. (**A**) 96-well plan for MCF7, MCF7-miR526b, and MCF7-miR655 cell lines. (**B**) 96-well plan for MCF7 cells treated with basal media or MCF7-miR526b/miR655 conditioned media. (**C**) 96-well plan for HUVECs treated with basal media or MCF7-miR526b/miR655 conditioned media. Negative controls were not included for HUVECs, because the toxicity of the ROS inducer had a drastic effect on HUVEC survival (Figure S4) compared to negative controls. Therefore, we calculated ROS/SO production by using basal emissions as the reference.

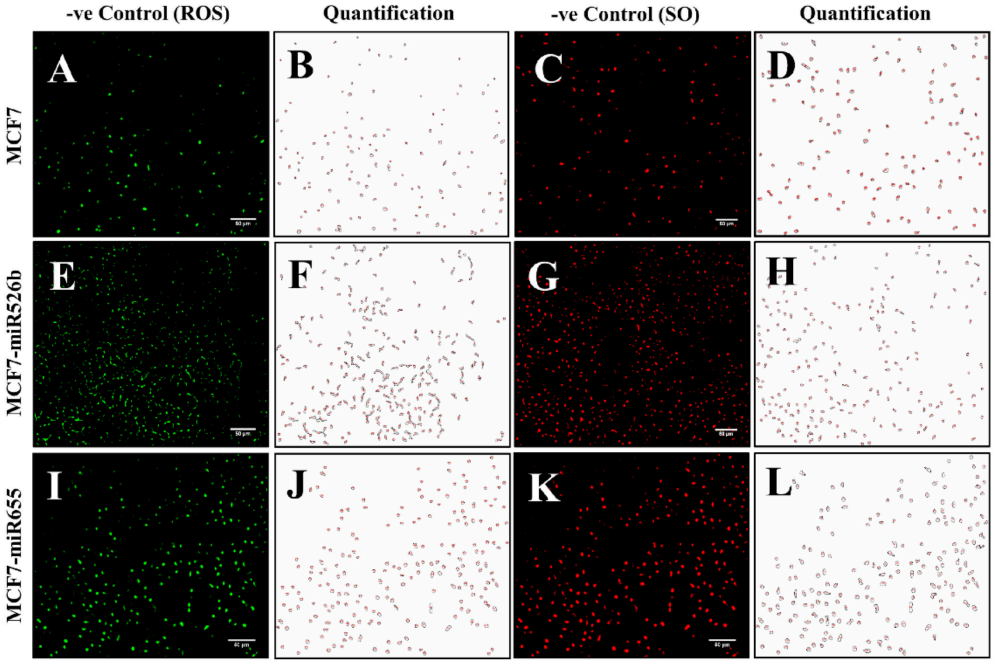

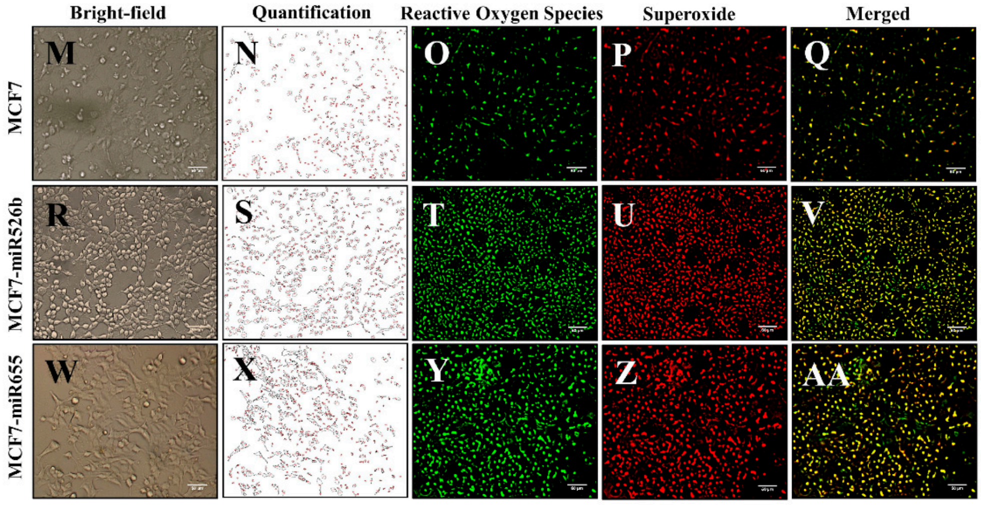

Figure S2. Fluorescence microscopy with MCF7, MCF7-miR526b, MCF7-miR655 using Fluorescein and Rhodamine filters. (A, E, I) Negative control ROS (green) fluorescence images of MCF7, MCF7-miR526b, and MCF7-miR655 cells, respectively. **(B, F, J)** Cell quantification of ROS negative control images for MCF7, MCF7-miR526b, and MCF7-miR655 cells, respectively. **(C, G, K)** Negative control SO (red) images of MCF7, MCF7-miR526b, and MCF7-miR655 cells, respectively. **(D, H, L)** Cell quantification of SO negative control images for MCF7, MCF7-miR526b, and MCF7-miR655 cells, respectively. **(M, R, W)** Bright-field images of MCF7, MCF7-miR526b, and MCF7-miR655 cells, respectively. **(N, S, X)** Cell quantification of bright-field images MCF7, MCF7-miR526b, and MCF7-miR655 cells, respectively. **(O, T, Y)** Fluorescence microscopy images of ROS (green) in MCF7, MCF7-miR526b, and MCF7-526b cells, respectively. **(P, U, Z)** Fluorescence microscopy images of SO (red) in MCF7, MCF7-miR526b and MCF7-526b cells respectively **(Q, V, AA)** Fluorescence microscopy images of ROS and SO merged (yellow) in MCF7, MCF7-miR526b and MCF7-526b cells respectively. Scale bar: 50µm.

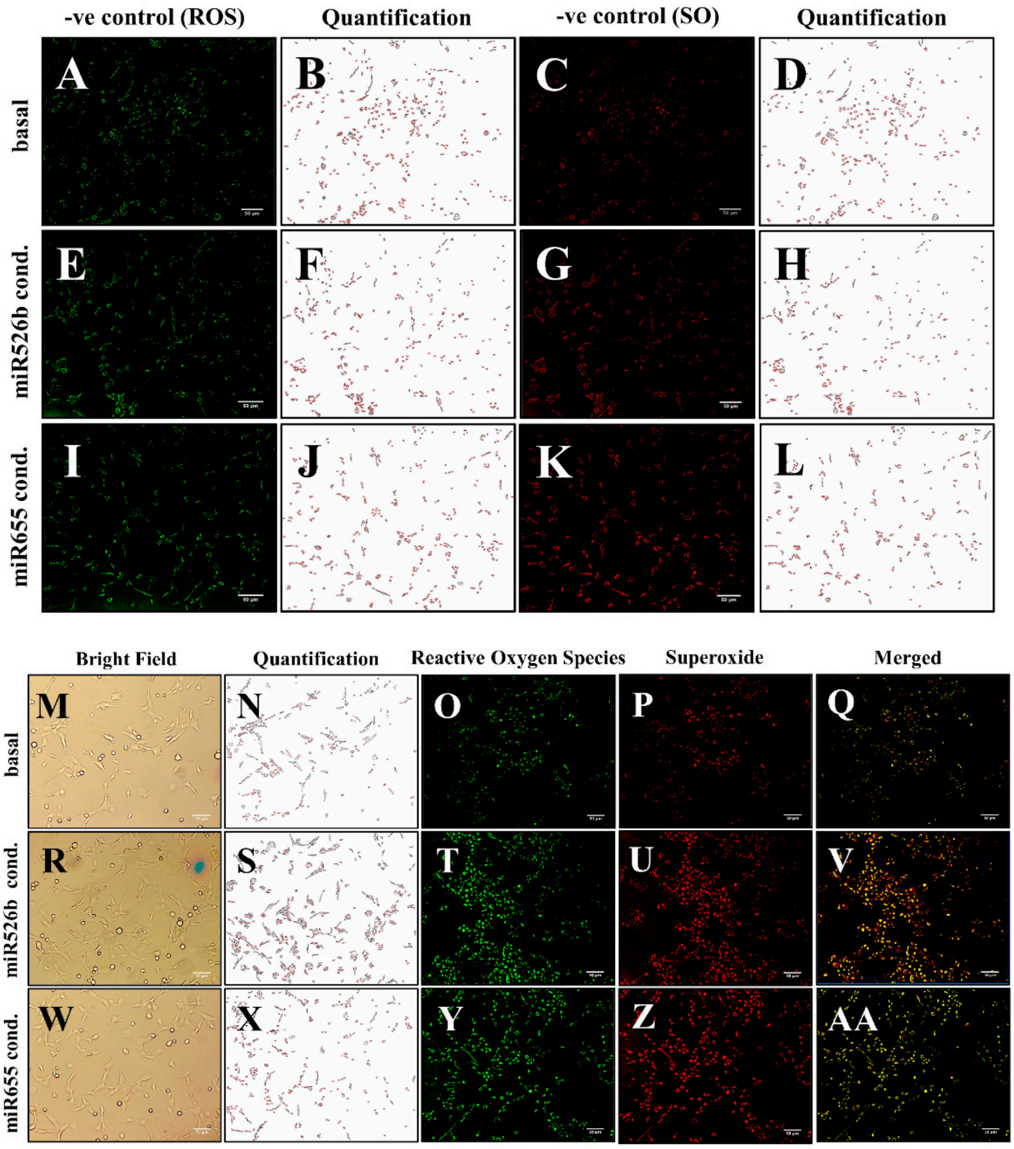

Figure S3. Fluorescence microscopy with MCF7 cells treated with basal media, MCF7-miR526b or MCF7-miR655 conditioned media using Fluorescein and Rhodamine filters. (A,E,I) Negative control images of ROS in MCF7 cells treated with basal media, MCF7-miR526b, and MCF7-miR655 conditioned media, respectively. **(B,F,J)** Cell quantification of ROS negative control images in MCF7 cells treated with basal media, MCF7-miR526b, and MCF7-miR655 conditioned media, respectively. **(C,G,K)** Negative control images of SO in MCF7 cells treated with basal media, MCF7-miR526b, and MCF7-miR655 conditioned media, respectively. **(D,H,L)** Cell quantification of SO negative control images in MCF7 cells treated with basal media, MCF7-miR526b, and MCF7-miR655 conditioned media, respectively. **(M,R,W)** Bright field images of MCF7 cells treated with basal media, MCF7-miR526b, and MCF7-miR655 conditioned media, respectively. **(N,S,X)** Cell quantification of bright field images of MCF7 cells treated with basal media, MCF7-miR526b, and MCF7-miR655 conditioned media. **(O,T,Y)** ROS (green) fluorescence images of MCF7 cells treated with basal media, MCF7-miR526b, and MCF7-miR655 conditioned media, respectively. **(P,U,Z)** SO (red) fluorescence images of MCF7 cells treated with basal media, MCF7-miR526b, and MCF7-miR655 conditioned media, respectively.

(Q,V,AA) Fluorescence images of ROS and SO merged (yellow) in MCF7 cells treated with basal media, MCF7-miR526b, and MCF7-miR655 conditioned media, respectively. Scale bar: 50µm.

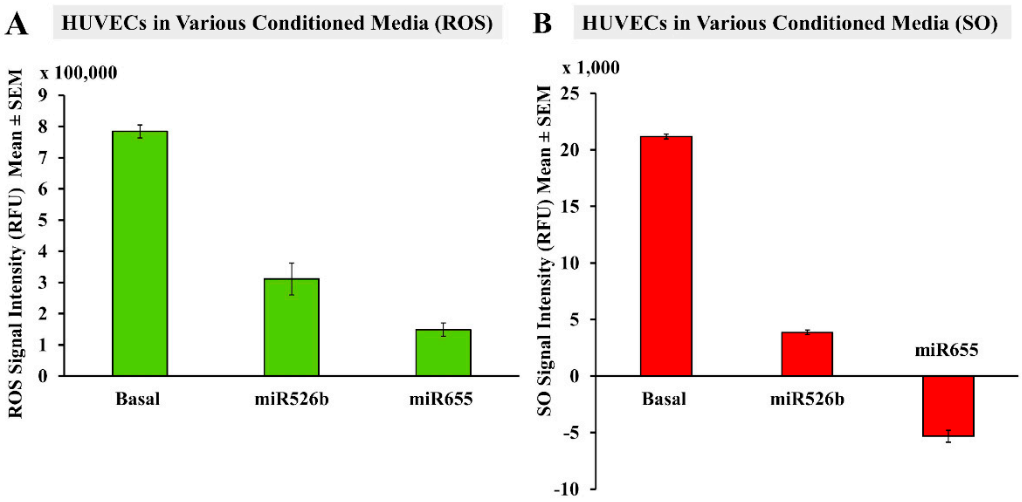

Figure S4. (A/B) Fluorescence Microplate assay with HUVECs treated with basal media or MCF7-miR526b/655 conditioned media 1 h after detection dyes were added; primary cells are more sensitive to the toxicity of cancer cells' media and ROS inducer than cancer cells, so after 1 h HUVECs start to die, leading to distrustful readings.
